# Demand starter data kit: Selected socio-economic and technical energy system demand modelling data for all 47 counties in Kenya

**DOI:** 10.1016/j.dib.2025.111556

**Published:** 2025-04-17

**Authors:** Neve Fields, Ariane Millot, Martin Mutembei, Anne Nganga, Pietro Lubello, Leonhard Hofbauer, Mark Howells, Ed Brown

**Affiliations:** a STEER Centre, Department of Geography and Environment, Loughborough University, Loughborough, United Kingdom; b Department of Chemical Engineering, Imperial College London, London, United Kingdom; c Strathmore Energy Research Centre (SERC), Strathmore University, Nairobi, Kenya; d Energy Institute, University College London, London, United Kingdom; e Centre for Environmental Policy, Imperial College London, London, SW7 1NE, United Kingdom

**Keywords:** U4RIA, MAED, Renewable energy, County energy planning, Energy systems, Data-to-deal, Energy policy

## Abstract

The need for data-driven models to inform energy planning and policy making is increasingly important as Kenya looks to transform its energy system to be clean, efficient, diverse and secure. Modelling softwares can be used by policy makers to assess the impacts of different scenarios on energy systems to support planning and decision making. Demand forms an integral foundation of energy planning and insights into possible projections can aid in policy creation, yet access to data is often a barrier to utilising energy demand modelling to support such decision making. Despite the official launch of the energy governance devolution process within Kenya, through the Kenya Energy Act (2019), progress towards county energy planning and developing modelling data and tools to reflect this remains limited and inaccessible. Therefore, this article provides data that can be used to create a simple whole energy system demand model for the individual counties in Kenya, acting as a starting point for teaching, capacity building efforts, and for further data collection, model development and scenario analysis to produce county resolution demand projections. The data was collected from websites, annual reports, and databases of international and national organisations alongside existing modelling databases and academic articles and can be easily updated based on the latest available local data and information. As a demonstration, these data were used to calibrate a demand model for Kilifi County using the Model for the Analysis of Energy Demand (MAED) for a baseline scenario from 2019 to 2070. The assumptions used and results gained are illustrated in the appendix of the article as a demonstration of what can be achieved through application of this dataset.

Specifications Table

**Table:** 

Subject	Earth & Environmental Sciences
Specific subject area	Energy System Modelling
Type of data	Table, Graph, Figure Raw, Analysed
Data collection	Data were collected via websites, annual reports and databases of international and national organisations alongside existing modelling databases and academic articles. Open and accessible data sources were preferred. The data were collected for use with the Model for the Analysis of Energy Demand (MAED) tool, which can project whole energy system demand based on historical data. Despite this, the data outlined through this document remains independent from the models and tools identified. Units were checked to be consist across all entries. The model period covered was 2019–2070.
Data source location	Collection methodology, raw data sources, and assumptions are listed within the article.
Data accessibility	With the article and in a repository. Repository name: Zenodo Data identification number: 10.5281/zenodo.14725145 Direct URL to data: https://zenodo.org/records/14725146
Related research article	None

## 1 Value of the Data

• Can be used to develop county energy system demand models to inform county energy plans, investment outlooks and policy, alongside providing insights on the evolution of energy system demand under varying trajectories.
• The socio-economic and technical data are useful for capacity building efforts, teaching, and consultation for country analysts, policymakers, and the broader scientific community. Additionally, enabling further development in Kenya of open and accessible evidence-informed energy model development and scenario creation, with the flexibility of MAED facilitating the addition of further end uses and sectors in the future such as transport, air conditioning and water heating. Nevertheless, the data gathered in this paper are independent of the tool.
• The data are open-source and county-specific, overcoming barriers to a lack of easily accessible data sets in current literature. By compiling the data from multiple diverse sources, the work provides analysts and academics with complete and accessible datasets needed to complete county level demand projections, overcoming barriers of data inaccessibility.
• The data is compiled into one repository, reducing time and financial resources necessary for data collection and therefore useful for capacity building efforts of a shorter time period.
• The dataset promotes the U4RIA principles [1], which are Ubuntu, Retrievability, Reusability, Repeatability, Reconstructability, Interoperability, and Auditability.
• The dataset can be used to support evidence informed interdisciplinary research across energy, development and nexus sectors, to examine trends and regional variations within Kenya, and to support future planning and policy making.

## 2 Background

Energy is widely heralded as an integral enabler of social and economic development globally [2]. Energy planning is critical to achieving successful energy transitions and electrification, and energy systems modelling is a widely applied tool to aid in the development evidence-informed policy making [3]. In Kenya, the government is already utilising a range of energy models to inform their planning procedures [4]. The Kenyan Energy Act of 2019 officially authorised the devolution of regulatory and policy functions for energy sector planning and decision making from the national to county governments [5]. This shift in energy governance procedures requires an update in energy modelling tools and research procedures to respond to this change. Alongside this, county energy departmental analytical, political and operational capacity requires significant development to effectively respond to their obligations. Within county energy planning units, access to data, time, resources and skills remain a major barrier to county energy plan (CEP) and county integrated development plan (CIDP) development, with only 6 counties having developed their CEPs and a further 15 additional counties working on their plans by April 2023[6].

The aim of this dataset is to bridge the current data gap by collating and providing key socio-economic and technical input data and assumptions for long-term energy demand forecasting and planning at the county resolution in Kenya, overcoming historic barriers to county energy research and planning of a lack of data availability and time and resource capacity constraints. The data provides a set of centralised and accessible county resolution data needed to conduct energy system demand research and can be used as inputs for whole energy system optimisation modelling studies. The dataset contains a novel production of county resolution energy balances and demand model application, providing the first set of ‘starter data’ for all 47 counties in Kenya.

The data is aimed at, and can be used by academics, policy makers, government officials, or consultants for further energy system modelling development and analysis. Additionally, it is designed to establish a foundation template of base data from which to develop further in-country modelling capacity and improve data accuracy through updating the data with bottom-up, on-the-ground data collected at the county level. To the best knowledge of the authors, a comprehensive dataset outlining Kenya county specific socio-economic and technical data for energy demand modelling, as well as a comprehensive outline of the assumptions made and why, does not currently exist in the public sphere. The article consists of entirely new data which has not yet previously been employed or analysed in existing research studies.

## 3 Data Description

This paper presents historical and projected socio-economic and technical data from 2019 to 2070 by county, and related national data, within Kenya and can be used as input data to develop county resolution demand projections using the Model for the Analysis of Energy Demand (MAED). MAED is a simple, easy to use, energy demand forecasting tool developed by the International Atomic Energy Agency (IAEA) that uses a bottom-up techno-economic simulation methodology [7]. The complete list of counties included within this dataset can be found in the appendix, with an example energy demand model; however, additional more comprehensive county-specific datasets are available externally for each county (see Appendix B for links to each available county-specific dataset to be consulted by analysts wanting to use the data for their own research). The time-period is selected based on data availability, for the base year of 2019, and relevancy to long-term energy planning time scales in national Kenyan energy planning. The data was collected via publically available sources, including the reports of national and international organisations, journal articles, and existing databases, complying with the U4RIA analytics and good governance goals of Ubuntu, Retrievability, Reusability, Repeatability, Reconstructability, Interoperability, and Auditability. The U4RIA goals aim to provide guidance on best practices within energy modelling [1]. The methods of data collection and preparation are outlined in Section 2 of this document. The datasets include raw data on gross county product (GCP), population alongside national energy consumption, energy efficiency and electrification rates where available. County energy consumption by sector (agriculture, construction, mining, manufacturing, services, and households) were processed based on assumptions. This disaggregation was constructed to align with the structure of the Model for the Analysis of Energy Demand (MAED) tool, however the data outline in this paper remains independent from the tool. For consistency and relevance, all economic data is presented in Kenyan Shillings and all energy data in terajoules (TJ). Additionally, a short data dictionary is provided for user ease (Table 1).

**Table 1:** A brief data dictionary of key terms throughout the dataset.

Key term	Description
Electrification	Percentage of a given population with access to electricity.
Energy Balance	A presentation of the total production, transformation and consumptions of different fuel types across different sectors across a geographically defined area (usually a country).
Energy Intensity	A unit of energy efficiency which is measured by the unit of energy required per unit of output.
Gross County Product (GCP)	Total monetary value of goods and services produced within a county.
Gross Domestic Product (GDP)	Total national monetary value of goods and services produced within a country.
Urban	The percentage of a given population residing in a municipality or town.
Urbanisation	The increase in the percentage of the total population residing in municipalities or towns, driven by rural to urban migration.

For comprehensive understanding and ease of use, county-specific datasets, in the format of a CVS, MAED model and associated PDF, are available externally for each county in a repository. All data is available in the CVS files, with a step-by-step methodology outlined in a PDF, for both individual counties and in a combined format. Cleaned datasets with all key variables in a structured format are available for the individual county files, in addition to the combined and aggregated raw data files. The data in the county demand starter data kit were collected from the KNBS data portal [8], economic [[9], [10], [11], [12]] and census [[13], [14], [15], [16], [17]] reports, the KIPPRA economic report [18], the International Energy Agency (IEA) world energy balances [19], the commission on revenue allocation county fact sheets [20] and previous modelling literature [21]. The datasets used were acquired from national and international energy organisations, who employ scientifically recognised and approved sampling methodologies, providing results which are likely to be representative of the overall economic and population conditions within Kenya, strengthening the reliability of the data utilised within this dataset. The Kenya National Bureau of Statistics (KNBS) is the primary agency of the Kenyan government, and is responsible for collecting, analysing and disseminating all statistical data in Kenya, employing rigorous standards and best practices. For the KNBS economic surveys, cluster sampling methodologies were employed to acquire data, alternatively for the KNBS census data stratified sampling was utilised (Table 2).

**Table 2:** A list of all the tables and figures containing summaries and illustrations of the data presented within the paper, alongside a description of their content.

Item	Description of content
Table 2	A brief data dictionary of key terms throughout the dataset.
Table 3	An overview of the input population parameters for the base year of 2019 including population, urban–rural population split, people per urban and rural household, and the urbanisation rate.
Fig. 1	An illustrative map of the population distribution across all 47 counties in Kenya.
Fig. 2	An illustrative map of the percentage of the population residing in urban centres across all 47 counties in Kenya.
Fig. 3	An illustrative map of the average household size across all 47 counties in Kenya.
Table 4	An overview of the collected population growth rate (%) projections for the individual counties across select years.
Table 5	An overview of the input economic parameters for the base year of 2019 including Gross County Product (GCP), GCP average growth rate, and GCP share for the sectors of agriculture, construction, mining, manufacturing, services and energy.
Fig. 4	An illustrative map of the average annual GCP growth rate across all 47 counties in Kenya.
Fig. 5	An illustrative map of the total GCP across all 47 counties in Kenya for 2019.
Table 6	An overview of the collected electrification rate (%) projections for the individual counties across select years.
Fig. 6	An illustrative map of the average annual electrification rates from 2009 to 2019 for all 47 counties in Kenya.
Table 7	National Energy Balance (TJ) for the base year of 2019, including energy consumption data for the agriculture, construction, mining, manufacturing, household and services sectors, disaggregated into the fuel types of fossil fuels, traditional fuels and electricity.
Table 8	County Sectoral contribution (%) to the total sectoral GDP for agriculture, construction, mining, manufacturing and services sectors, and population for the household sector.
Table 9	County Energy Balance (TJ) for the base year of 2019, including aggregated energy consumption data for the agriculture, construction, mining, manufacturing, household and services sectors.
Table 10	Derived energy intensity figures for each fuel type (motive, electricity, and thermal) per sector (agriculture, construction, mining, manufacturing, and services) per county.
Table 11	Derived energy intensity figures for urban and rural households, per end use (cooking lighting and appliances) per fuel type (fossil fuels and appliances).
Fig. 7	An illustrative representation of the downscaling methodology employed to acquire county resolution energy balance data from raw national energy balance data using the example of household and manufacturing sectors for Kilifi.
Fig. 8	An illustrative overview of the demand structure employed in the Model of the Analysis of Energy Demand (MAED).
Fig. 9	An illustrative overview of the MAED energy intensity structure for individual energy end uses per sector.

### 3.1 Population

**Fig. 1:**
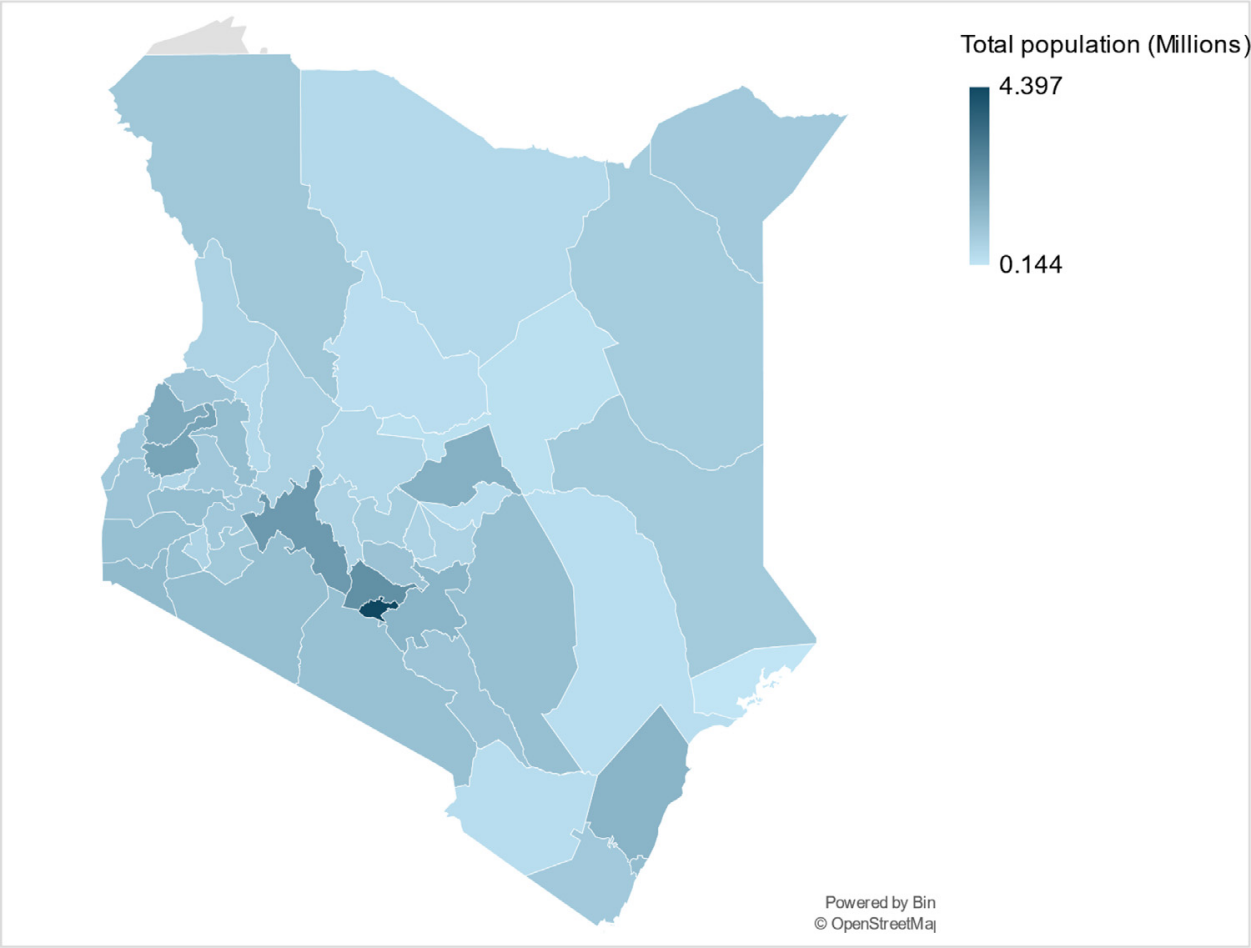
An illustrative map of the population distribution across all 47 counties in Kenya. Note that the population density is mapped as total population (million people) per Kenyan county.

**Fig. 2:**
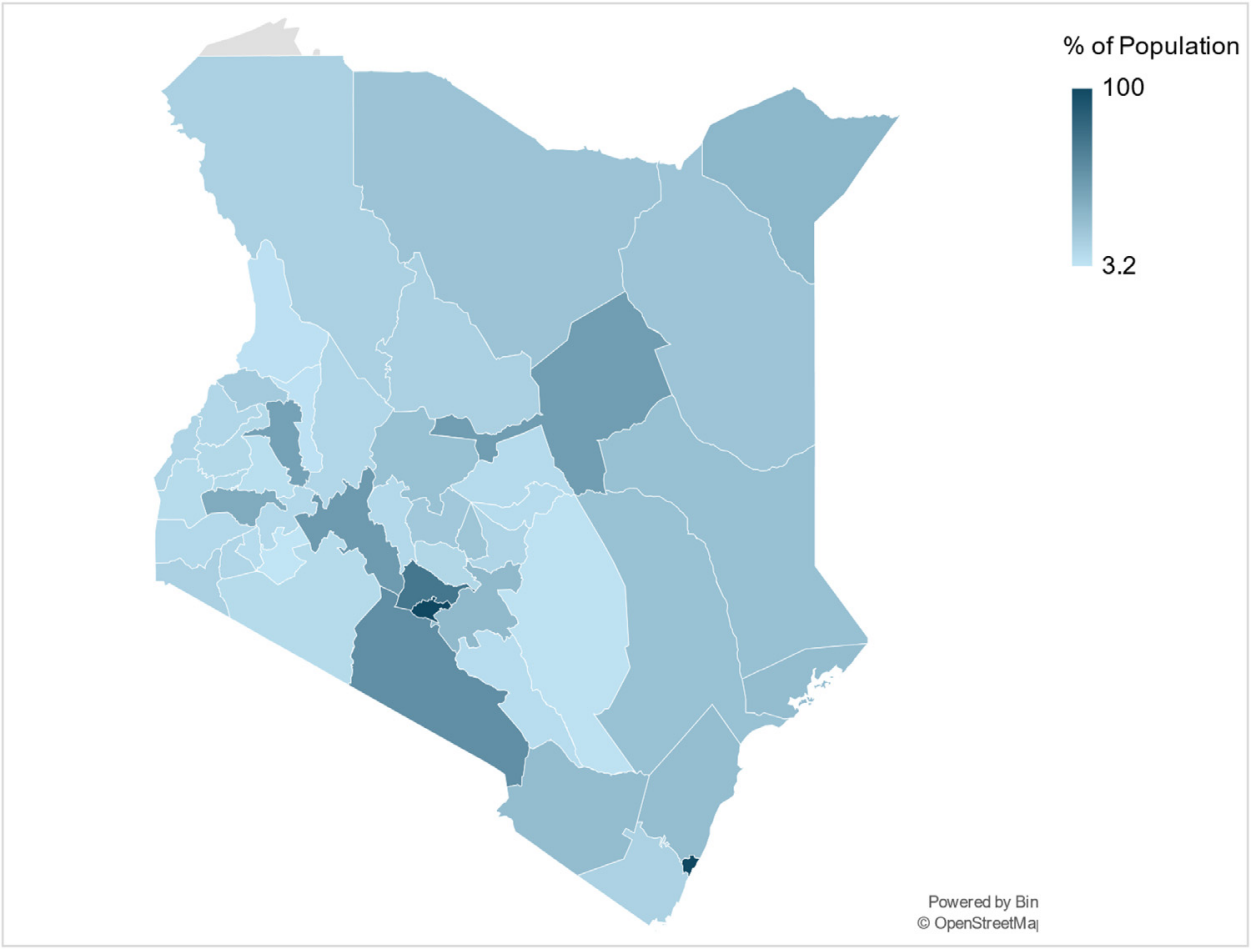
An illustrative map of the percentage of the population residing in urban centres across all 47 counties in Kenya.

**Fig. 3:**
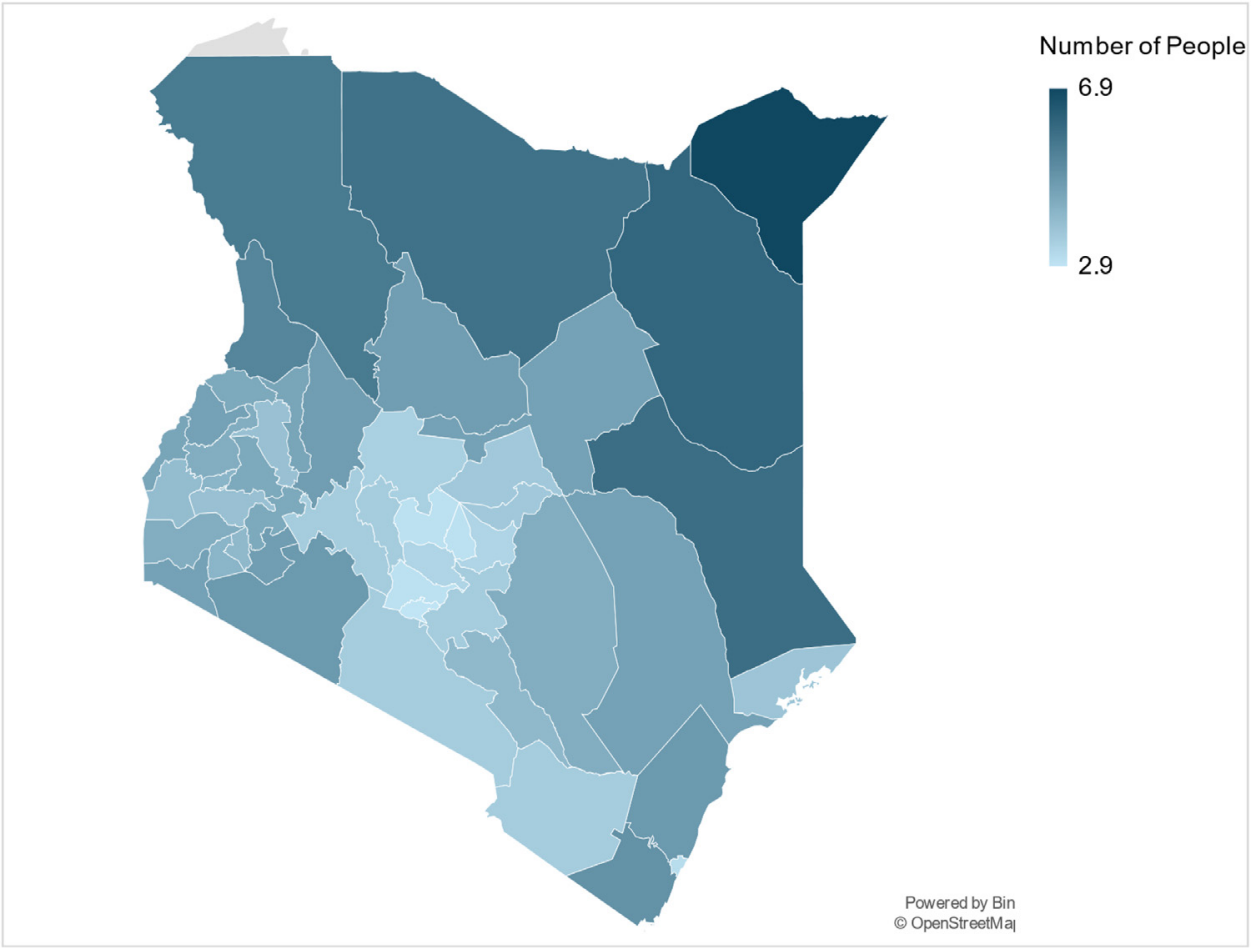
An illustrative map of the average household size across all 47 counties in Kenya. Note that the average household size is mapped as average number of people per household per Kenyan county.

Various population data and parameters can be used to reconstruct base years, and acquire the driving parameters necessary to project energy demand using energy models, presented in Table 2, Table 3. Collected population data include total population (million people), population growth rates (%), people per household, population share by urban and rural (%), and urbanisation rate (%) for the counties are presented in the dataset, for the base year of 2019. Data was collected from the 2019 national census [15], the world bank database [22], and the KNBS data portal [8]. The assumptions made in the population data are described in more detail in the methods section. The inputted data for each individual year from 2019 to 2070 can be found in the county datasets published on Zenodo (see Appendix B). Using the dataset, various population trends can be analysed. For example, understanding regional, and county, variations in population distribution (Fig. 1) and urbanisation (Fig. 2), alongside regional differences in average household size (Fig. 3) and how this could impact household energy usages across the region.

**Table 3:** An overview of the input population parameters for the base year of 2019 including population, urban-rural population split, people per urban and rural household, and the urbanisation rate.

County	Population (Million)	Urban Population %	Person/ Urban Household	Rural Population %	Person/ Rural Household	Urbanisation Rate %
Baringo	0.667	11.3	4.7	88.7	4.7	0.5
Bomet	0.902	3.2	4.7	96.8	4.7	0.5
Bungoma	1.671	11.4	4.6	88.6	4.6	0.5
Busia	0.894	12.7	4.5	87.3	4.5	0.5
Elgeyo Marakwet	0.454	4.5	4.5	95.5	4.5	0.5
Embu	0.608	12.5	3.3	87.5	3.3	0.5
Garissa	0.841	25.1	5.9	74.9	5.9	0.5
Homa Bay	1.132	10	4.3	90	4.3	0.5
Isiolo	0.268	46.9	4.6	53.1	4.6	0.5
Kajiado	1.118	55.7	3.5	44.3	3.5	0.5
Kakamega	1.867	9.9	4.3	90.1	4.3	0.5
Kericho	0.876	10.4	4.4	89.6	4.4	0.5
Kiambu	2.418	70.6	3	29.4	3	0.5
Kilifi	1.454	27.1	4.8	72.9	4.8	0.5
Kirinyaga	0.61	22.3	3	77.7	3	0.5
Kisii	1.116	12	4.1	88	4.1	0.5
Kisumu	1.156	38.2	3.8	61.8	3.8	0.5
Kitui	1.136	4.8	4.3	95.2	4.3	0.5
Kwale	0.867	14.6	5	85.4	5	0.5
Laikipia	0.519	24.6	3.4	75.4	3.4	0.5
Lamu	0.144	27.4	3.7	72.6	3.7	0.5
Machakos	1.422	29.1	3.5	70.9	3.5	0.5
Makueni	0.988	7.8	4	92.2	4	0.5
Mandera	0.867	31.2	6.9	68.8	6.9	0.5
Marsabit	0.459	23.3	5.8	76.7	5.8	0.5
Meru	1.546	9	3.6	91	3.6	0.5
Migori	1.267	15	4.6	85	4.6	0.5
Mombasa	1.208	100	3.1	-	-	0.5
Murang’a	1.057	11.2	3.3	88.8	3.3	0.5
Nairobi	4.397	100	2.9	-	-	0.5
Nakuru	2.162	48.4	3.5	51.6	3.5	0.5
Nandi	0.886	6.7	4.4	93.3	4.4	0.5
Narok	1.158	8.7	4.8	91.3	8.7	0.5
Nyamira	0.606	8.3	4	91.7	4	0.5
Nyandarua	0.638	10.4	3.5	89.6	3.5	0.5
Nyeri	0.759	19.9	3	80.1	3	0.5
Samburu	0.31	15.2	4.7	84.8	4.7	0.5
Siaya	0.993	8.6	3.9	91.4	3.9	0.5
Taita Taveta	0.341	27.5	3.5	72.5	3.5	0.5
Tana River	0.316	24.7	4.6	75.3	4.6	0.5
Tharaka Nithi	0.393	8.3	3.6	91.7	3.6	0.5
Trans Nzoia	0.99	18	4.4	82	4.4	0.5
Turkana	0.927	15.2	5.6	84.8	5.6	0.5
Uasin-Gishu	1.163	43.9	3.8	56.1	3.8	0.5
Vihiga	0.59	9.9	4.1	90.1	4.1	0.5
Wajir	0.781	22.7	6.1	77.3	6.1	0.5
West Pokot	0.621	5.1	5.3	94.9	5.3	0.5

### 3.2 Economic

**Fig. 4:**
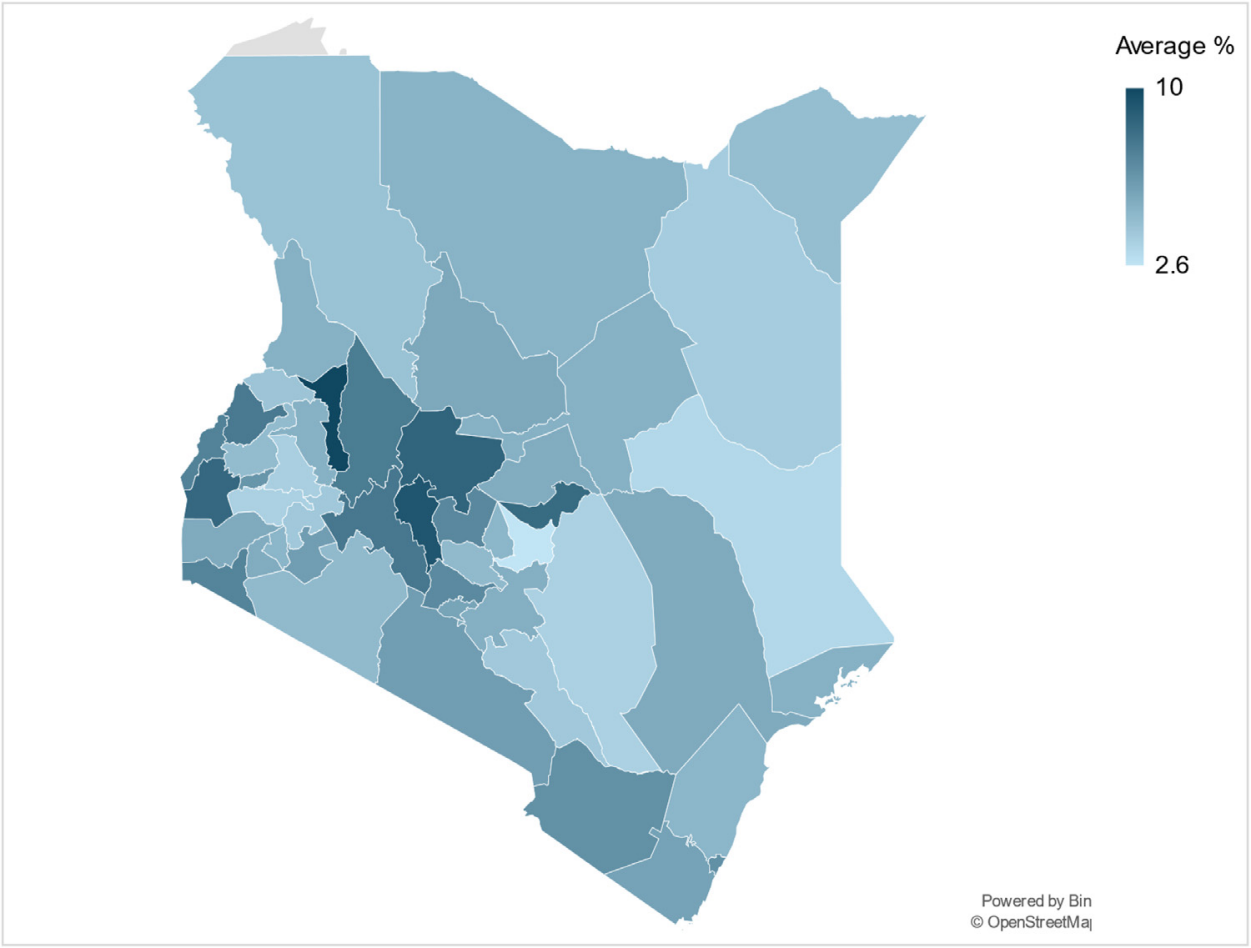
An illustrative map of the average annual GCP growth rate across all 47 counties in Kenya. Note that total GCP is presented as Billion Kenyan Shillings.

**Fig. 5:**
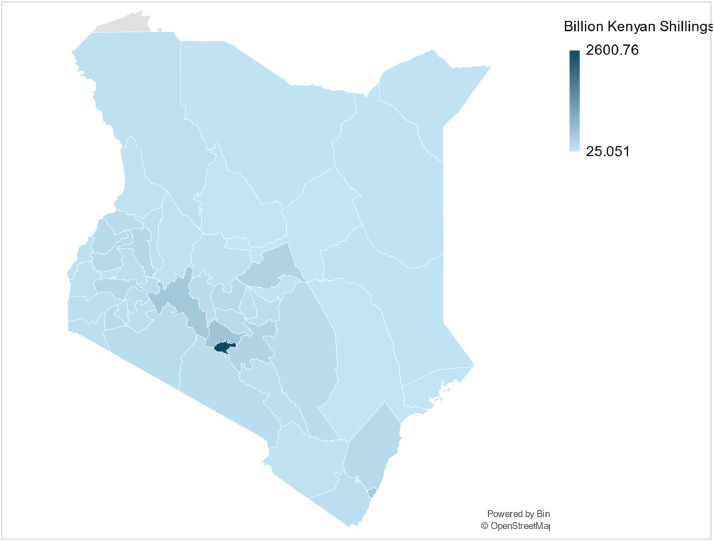
An illustrative map of the total GCP across all 47 counties in Kenya for 2019.

Numerous economic data and parameters can be used alongside population data to reconstruct base years and acquire the driving parameters necessary to project energy demand using energy models, presented in Table 4. Collated economic data includes total gross county product (GCP) (Billion Kenyan Shillings), GCP growth rate (%), and sectoral GCP share splits (across agriculture, construction, mining, manufacturing, services and energy) for the base year of 2019. Data was collected from the 2020 KIPPRA economic report [18], the 2021 [11] and the 2023 [10] KNBS gross county product report [11]. The assumptions made in the population data are described in more detail in the methods section. The inputted data for each individual year from 2019 to 2070 can be found in the county datasets published on Zenodo (see Appendix B). Economic trends such as county disparities in economic growth (Fig. 4) and differences in economic configurations (Fig. 5) can be analysed through utilising this dataset.

**Table 4:** An overview of the collected population growth rate (%) projections for the individual counties across select years.

County	2020	2030	2040	2050	2060	2070
Baringo	2.993	1.841	1.318	1.033	0.937	0.856
Bomet	−0.264	1.111	0.811	0.655	0.615	0.579
Bungoma	1.786	1.284	0.912	0.739	0.689	0.644
Busia	2.228	1.667	1.273	1.039	0.941	0.86
Elgeyo Marakwet	4.387	1.226	0.913	0.719	0.67	0.628
Embu	3.274	0.887	0.669	0.561	0.531	0.504
Garissa	2.359	1.9907	1.482	1.2089	1.0785	0.9735
Homa Bay	2.643	1.791	1.469	1.207	1.077	0.972
Isiolo	9.739	2.129	1.809	1.5001	1.304	1.154
Kajiado	5.449	2.036	1.616	1.347	1.187	1.061
Kakamega	1.588	1.494	1.113	0.914	0.837	0.773
Kericho	4.742	1.113	0.839	0.691	0.646	0.607
Kiambu	3.444	1.705	1.377	1.178	1.054	0.954
Kilifi	2.393	1.7004	1.401	1.182	1.057	0.956
Kirinyaga	4.378	0.773	0.636	0.5401	0.512	0.487
Kisii	17.043	0.752	0.489	0.393	0.378	0.364
Kisumu	2.647	1.452	1.154	0.972	0.886	0.814
Kitui	4.388	1.043	0.784	0.629	0.592	0.558
Kwale	1.414	2.28	2.047	1.697	1.451	1.267
Laikipia	1.919	1.796	1.475	1.227	1.093	0.985
Lamu	7.542	2.347	2.073	1.719	1.467	1.279
Machakos	1.392	0.839	0.585	0.475	0.453	0.434
Makueni	2.012	1.004	0.832	0.698	0.653	0.613
Mandera	2.285	2.382	1.944	1.578	1.363	1.199
Marsabit	4.305	2.198	1.771	1.405	1.232	1.097
Meru	1.275	1.132	0.901	0.749	0.697	0.651
Migori	−9.446	2.156	1.787	1.479	1.289	1.142
Mombasa	1.634	1.852	1.415	1.169	1.046	0.947
Murang’a	1.883	0.978	0.785	0.664	0.623	0.586
Nairobi	2.696	1.38	1.049	0.868	0.798	0.739
Nakuru	1.833	1.853	1.464	1.216	1.084	0.978
Nandi	2.249	1.389	1.054	0.865	0.796	0.737
Narok	1.714	2.532	1.999	1.603	1.382	1.214
Nyamira	6.599	0.505	0.374	0.303	0.295	0.286
Nyandarua	2.956	1.615	1.24	1.036	0.939	0.858
Nyeri	6.643	0.947	0.745	0.6404	0.602	0.568
Samburu	3.216	2.585	2.184	1.755	1.493	1.299
Siaya	0.982	1.675	1.379	1.155	1.035	0.938
Taita Taveta	2.919	1.109	0.979	0.841	0.775	0.719
Tana River	3.143	2.439	2.206	1.822	1.541	1.335
Tharaka Nithi	2.524	0.919	0.697	0.595	0.561	0.531
Trans Nzoia	2.012	1.531	1.777	0.98	0.893	0.819
Turkana	2.102	2.401	1.9803	1.591	1.373	1.207
Uasin-Gishu	1.706	1.728	1.384	1.149	1.031	0.934
Vihiga	3.375	0.732	0.537	0.447	0.428	0.411
Wajir	2.895	2.414	1.972	1.573	1.359	1.197
West Pokot	1.591	2.207	1.902	1.553	1.344	1.185

### 3.3 Electrification

**Fig. 6:**
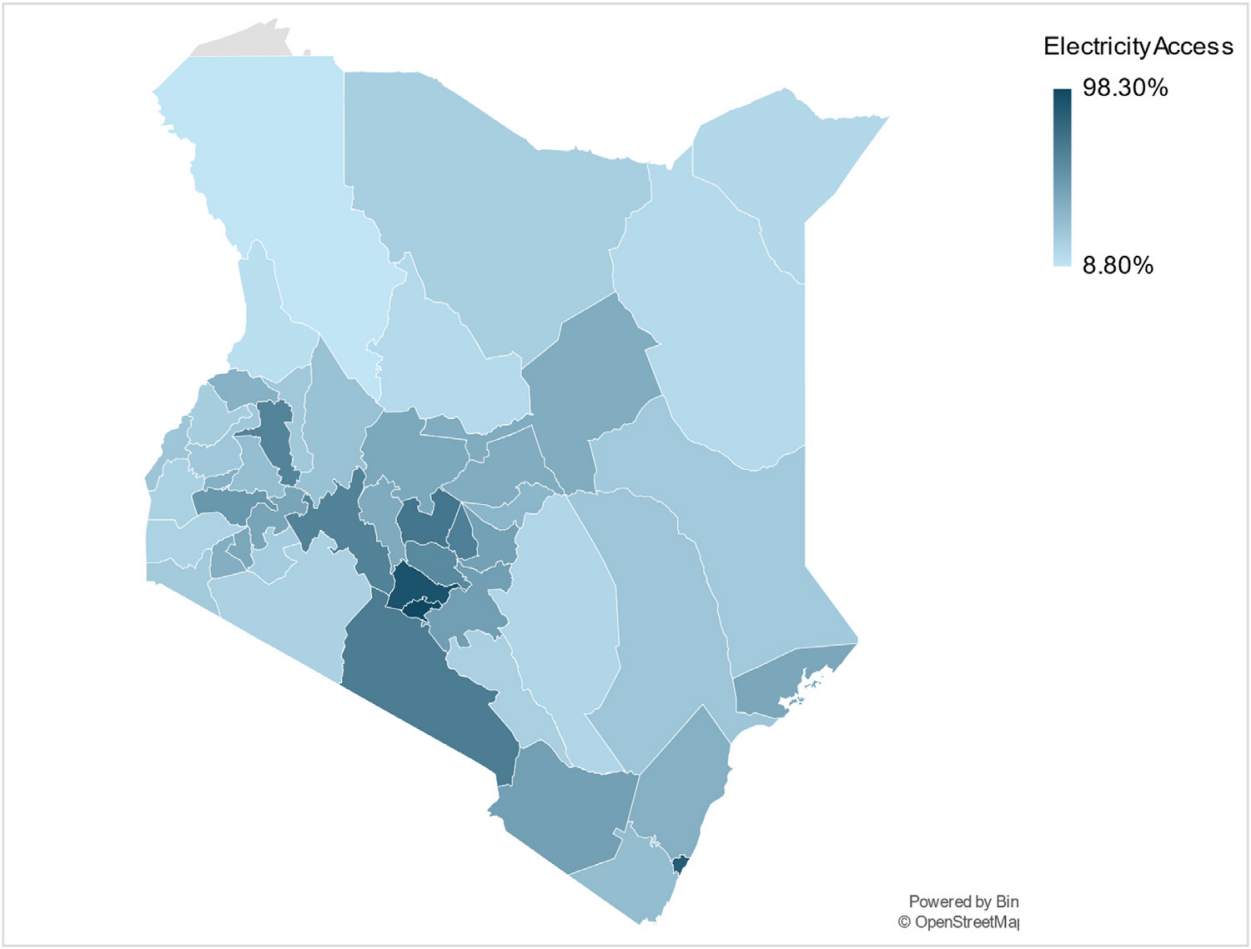
An illustrative map of the average annual electrification rates from 2009 to 2019 for all 47 counties in Kenya.

Electrification data can be used as an input parameter alongside population data to reconstruct the base year and acquire the driving parameters necessary to project energy demand for the household sectors using energy models (Table 5). Data on the base year (2019) electrification rates, and average yearly electrification increase, of the individual counties were obtained via the Commission of Revenue Allocations Kenya County fact sheets third edition [20]. The assumptions made in the population data are described in more detail in the methods section. The inputted data for each individual year from 2019 to 2070 can be found in the county datasets published on Zenodo (see Appendix B). The dataset can be used for further analysis on regional, and county, disparities in progress to universal electrification alongside differences in average annual progress to increased electricity access (Fig. 6).

**Table 5:** An overview of the input economic parameters for the base year of 2019 including Gross County Product (GCP), GCP average growth rate, and GCP share for the sectors of agriculture, construction, mining, manufacturing, services and energy.

County	Gross County Product	GCP Growth Rate	Agriculture GCP Share	Construction GCP Share	Mining GCP Share	Manufacturing GCP Share	Services GCP Share	Energy GCP Share
	Ksh Billion	%						
Baringo	70.754	7.5	34.38	2.36	0.19	5.96	56.94	0.17
Bomet	130.299	5.9	53.89	7.27	0.49	7.18	30.88	0.29
Bungoma	192.038	7.6	41.86	2.65	0.17	3.88	51.11	0.33
Busia	82.172	7.2	34.85	8.77	0.38	3.22	52.53	0.25
Elgeyo Marakwet	104.651	10	66.46	0.93	0.5	2.23	29.75	0.13
Embu	138.049	2.6	26.41	7.98	0.18	2.24	51.13	12.06
Garissa	54.178	3.2	28.64	3.57	1.08	2.27	63.61	0.83
Homa Bay	113.595	5.3	41.5	1.96	0.4	4.38	51.6	0.16
Isiolo	25.051	5	16.58	9.92	0.17	1.76	70.58	0.99
Kajiado	143.156	5.9	13.49	8.56	2.09	5.28	69.2	1.38
Kakamega	203.238	4.5	38.47	1.55	0.77	6.86	52.04	0.31
Kericho	151.418	3.9	44.79	2.88	0.1	7.19	43.99	1.05
Kiambu	533.771	6.8	16	14.65	0.84	12.9	53.67	1.94
Kilifi	196.456	4.8	19.95	0.99	0.81	18.82	58.91	0.52
Kirinyaga	116.133	4.8	45.41	0.49	1.25	4.28	48.1	0.47
Kisii	184.198	5.2	41.95	3.29	0.24	2.96	51.28	0.28
Kisumu	235.001	3.5	16.19	6.49	0.74	11.48	62.95	2.15
Kitui	141.24	3.5	33.03	2.32	0.54	3.12	60.91	0.08
Kwale	108.204	5.7	31.86	3.83	9.1	5.67	49.16	0.38
Laikipia	88.434	8.6	26.52	8.74	0.14	2.83	61.32	0.45
Lamu	32.191	5	29.4	0.42	0.42	3.75	65.72	0.29
Machakos	295.593	5.1	16.63	7.61	3.51	20.99	50.18	1.08
Makueni	108.701	3.9	27.64	3.85	0.63	4.48	63.21	0.19
Mandera	49.834	4.4	34.73	6.6	0.37	1.99	55.52	0.79
Marsabit	56.341	4.9	22.57	18.38	0.14	1.05	36.11	21.75
Meru	306.874	5.2	54.92	1.97	1.08	4.87	36.62	0.54
Migori	114.146	7.1	38.27	0.98	5.68	2.96	51.77	0.34
Mombasa	471.34	6.5	0.73	11.19	0.26	16.85	68.38	2.59
Murang’a	180.485	4.6	48.2	5.69	1.33	4.12	39.64	1.02
Nairobi	2600.76	5.6	0.2	9.67	0.03	11.8	77.28	1.02
Nakuru	461.937	7.7	23.87	4.98	0.14	7.03	52.9	11.08
Nandi	138.369	3.6	56.94	0.99	0.49	5.8	35.49	0.32
Narok	156.231	4.6	47.68	0.9	0.15	4.83	46.22	0.22
Nyamira	107.145	4.8	48.61	3.29	1.38	4.57	41.86	0.29
Nyandarua	140.461	9.3	69.58	0.43	0.54	1.45	27.8	0.2
Nyeri	193.368	6.9	37.28	0.95	1.25	6.54	53.49	0.49
Samburu	27.955	5.4	17.75	3.15	0.18	4.33	74.37	0.22
Siaya	98.071	8.4	36.57	5.93	1.24	3.14	52.65	0.47
Taita Taveta	60.82	6.5	22.96	4.55	1.29	4.11	66.72	0.37
Tana River	27.172	5.3	32.51	0.55	0.22	2.3	64.15	0.27
Tharaka Nithi	55.874	8.3	32.26	7.96	0.34	4.91	54.33	0.2
Trans Nzoia	151.079	4.1	39.6	2.32	0.19	3.59	54.05	0.25
Turkana	101.579	4.2	31.86	4.02	1.37	2.05	60.655	0.045
Uasin-Gishu	223.831	5	24.37	4.36	0.34	5.33	64.95	0.65
Vihiga	76.884	6.3	41.16	6.91	2.31	1.73	47.51	0.38
Wajir	46.964	3.7	34.64	8.73	1.02	2.44	53.05	0.12
West Pokot	68.35	5	37.16	0.84	4.46	1.29	50.3	5.95

### 3.4 Energy balances

Energy balance data is critical to acquiring the energy intensity figures necessary to reconstruct base year energy consumption and project future energy demand for all sectors. Data on the national energy balance for the base year of 2019 was extracted from the International Energy Agency (IEA) world extended energy balance dataset 2021 [19]. The national energy balance sector data was disaggregated to the sectors of agriculture, construction, mining, manufacturing, services and households using the KIPPRA Kenya economic report 2020 [18]. Similarly, the fuel types consumed were aggregated to the categories of fossil fuels, traditional fuels and electricity in line with energy demand model structures (Table 6). The county sectoral contribution (%) to the total national sectoral GDP and national population was obtained from the KNBS 2021 Gross County Report [11] and the Commission of Revenue Allocations Kenya County fact sheets third edition [20]. Subsequent extracted county energy balances for 2019 derived from the national energy balance (Table 6) and the county sectoral contributions (Table 7) is outlined in Table 8. The assumptions made to extract the national energy balance into demand model input format, and to extract county level energy balances is outlined in further detail in the methods section. The derived national and county energy balances in more detail can be found in the county datasets published on Zenodo (see Appendix B).

**Table 6:** An overview of the collected electrification rate (%) projections for the individual counties across select years.

County	Electrification Rate annual increase %	2019	2030	2040	2050	2060	2070
Baringo	1.91	28.7	35.83	43.89	53.76	65.86	80.68
Bomet	1.82	22.1	27.56	33.77	41.36	50.67	62.07
Bungoma	1.73	21.8	48.32	59.19	72.51	88.82	100
Busia	2.02	26.2	32.74	40.11	49.13	60.19	73.73
Elgeyo Marakwet	1.75	24.7	30.79	37.71	46.2	56.59	69.32
Embu	3.24	47.3	59.82	73.28	89.76	100	100
Garissa	1.24	24	29.76	36.46	44.66	54.71	67.02
Homa Bay	1.51	18.5	23	28.18	34.52	42.29	51.8
Isiolo	2.21	40.6	50.83	62.27	76.28	93.44	100
Kajiado	2.81	67.9	85.01	100	100	100	100
Kakamega	1.96	25.2	31.47	38.56	47.23	57.86	70.87
Kericho	3.43	45	57.14	70	85.75	100	100
Kiambu	3.78	91.9	100	100	100	100	100
Kilifi	2.19	38.6	48.32	59.19	72.51	88.82	100
Kirinyaga	4.96	66	84.86	100	100	100	100
Kisii	3.14	39.5	49.91	61.13	74.89	91.74	100
Kisumu	3.45	52.8	66.91	81.96	100	100	100
Kitui	1.24	17.2	21.33	26.67	32.01	39.21	48.03
Kwale	2.05	31.1	38.88	47.63	58.34	71.47	87.54
Laikipia	2.5	42.7	53.61	65.68	80.45	98.55	100
Lamu	2.66	43.6	54.83	67.17	82.28	100	100
Machakos	3.12	48.2	60.89	74.58	91.37	100	100
Makueni	1.45	20.4	25.35	31.06	38.04	46.6	57.09
Mandera	1.33	15.8	19.61	24.02	29.43	36.05	44.16
Marsabit	1.37	21.2	26.33	32.25	39.5	48.39	59.28
Meru	2.7	40.6	51.08	62.57	76.65	93.89	100
Migori	1.82	24	29.93	36.67	44.92	55.03	67.41
Mombasa	2.73	86.3	100	100	100	100	100
Murang’a	4.66	60.5	77.57	95.02	100	100	100
Nairobi	1.94	98.3	100	100	100	100	100
Nakuru	3.03	64.3	81.15	99.41	100	100	100
Nandi	2.43	30.7	38.52	47.19	57.8	70.81	86.74
Narok	1.4	19.9	24.72	30.28	37.09	45.44	55.66
Nyamira	3.74	43.2	54.9	67.25	82.38	100	100
Nyandarua	3.01	41.6	52.49	64.3	78.77	96.49	100
Nyeri	4.569	72	92.23	100	100	100	100
Samburu	0.84	14.6	18.03	22.09	27.06	33.15	40.61
Siaya	1.53	19.6	24.38	29.86	36.58	44.81	54.89
Taita Taveta	3.3	48	60.74	74.4	91.14	100	100
Tana River	2.36	26.1	32.73	40.089	49.11	60.158	73.69
Tharaka Nithi	2.69	35.3	44.41	54.4	66.63	81.63	99.99
Trans Nzoia	2.92	38.1	48.03	58.84	72.08	88.3	100
Turkana	0.64	8.8	10.85	13.29	16.28	19.94	24.43
Uasin-Gishu	3.6	63.9	81.09	99.34	100	100	100
Vihiga	3.16	38.6	48.78	59.75	73.2	89.66	100
Wajir	1.12	14.6	18.09	22.15	27.14	33.24	40.72
West Pokot	0.93	11.9	14.71	18.02	22.08	27.05	33.13

**Table 7:** National Energy Balance (TJ) for the base year of 2019, including energy consumption data for the agriculture, construction, mining, manufacturing, household and services sectors, disaggregated into the fuel types of fossil fuels, traditional fuels and electricity.

2019 National Energy Balance TJ				
	Fossil Fuels	Traditional Fuels	Electricity	Total
Agriculture	434.67	-	-	434.67
Construction	2819.232	-	1480.527	4299.759
Mining	313.248	-	164.503	477.751
Manufacturing	6331.32	-	57.61	6388.93
Household	1260.57	50044.1	1099.17	52403.84
Services	-	-	489.26	489.26
Total	11159.04	50044.1	3291.07	64494.21

**Table 8:** County Sectoral contribution (%) to the total sectoral GDP for agriculture, construction, mining, manufacturing and services sectors, and population for the household sector.

County Sectoral Contribution % to total GDP & Population						
County	Agriculture	Construction	Mining	Manufacturing	Services	Household
Baringo	1.133	0.271	0.187	0.528	0.714	1.441
Bomet	3.418	1.629	0.922	1.224	0.749	1.949
Bungoma	3.735	0.835	0.441	0.931	1.741	3.611
Busia	1.337	1.188	0.439	0.332	0.768	1.932
Elgeyo Marakwet	3.209	0.159	0.722	0.289	0.547	0.982
Embu	1.689	1.803	0.338	0.385	1.533	1.316
Garissa	0.725	0.319	0.812	0.154	0.619	1.819
Homa Bay	2.199	0.367	0.634	0.624	1.0404	1.316
Isiolo	0.192	0.405	0.059	0.055	0.314	0.579
Kajiado	0.889	1.994	4.091	0.935	1.766	2.416
Kakamega	3.613	0.514	2.144	1.732	1.865	4.037
Kericho	3.145	0.714	0.217	1.356	1.199	1.893
Kiambu	3.925	12.692	6.113	8.501	5.174	5.226
Kilifi	1.83	0.323	4.957	4.64	2.033	3.143
Kirinyaga	2.587	0.1005	2.118	0.656	1.049	1.319
Kisii	3.576	0.992	0.6103	0.678	1.667	2.413
Kisumu	1.779	2.521	2.432	3.391	2.713	2.498
Kitui	1.941	0.481	0.944	0.492	1.359	2.456
Kwale	1.594	0.677	13.592	0.763	0.941	0.187
Laikipia	1.081	1.258	0.169	0.309	0.955	1.121
Lamu	0.438	0.022	0.187	0.15	0.373	0.311
Machakos	2.276	3.683	14.334	7.728	2.663	3.074
Makueni	1.392	0.685	0.948	0.606	1.212	2.135
Mandera	0.817	0.548	0.264	0.126	0.502	1.875
Marsabit	0.589	1.696	0.107	0.073	0.573	0.994
Meru	7.996	1.014	4.676	1.905	2.052	3.341
Migori	2.023	0.183	8.954	0.421	1.045	2.739
Mombasa	0.158	8.553	1.681	9.796	5.822	2.612
Murang’a	4.014	1.676	3.313	0.923	1.284	2.284
Nairobi	0.234	40.699	1.123	37.798	35.389	9.505
Nakuru	5.075	3.742	0.884	4.018	5.16	4.674
Nandi	3.686	0.228	0.881	1.009	0.879	1.915
Narok	3.444	0.2301	0.316	0.937	1.273	2.503
Nyamira	2.424	0.5803	2.047	0.612	0.798	1.309
Nyandarua	4.511	0.099	1.054	0.253	0.688	1.379
Nyeri	3.325	0.301	3.333	1.567	1.826	1.641
Samburu	0.23	0.144	0.0701	0.151	0.367	0.671
Siaya	1.669	0.955	1.689	0.385	0.919	2.147
Taita Taveta	0.644	0.451	1.082	0.309	0.714	0.736
Tana River	0.414	0.025	0.084	0.079	0.311	0.683
Tharaka Nithi	0.836	0.729	0.267	0.342	0.536	0.849
Trans Nzoia	2.765	0.572	0.406	0.674	1.438	2.141
Turkana	1.511	0.704	1.937	0.262	1.089	2.004
Uasin-Gishu	2.515	1.5901	1.059	1.479	2.568	2.514
Vihiga	1.48	0.878	2.478	0.167	0.653	1.275
Wajir	0.757	0.674	0.668	0.143	0.441	1.689
West Pokot	1.176	0.094	4.213	0.109	0.675	1.343
Total	100	100	100	100	100	100

### 3.5 Energy intensities

Energy efficiency data, expressed in the form of energy intensities as MJ/Kenyan Shilling or MJ/Dwelling/year for the different sectors and energy end use was derived using the population and economic driving parameter data outlined in Table 2, Table 3, Table 4, Table 5 and the county resolution energy balance data outlined in Table 8. The obtained energy intensity figures per energy end use per sector are outlined in Table 9, Table 10. The assumptions made to derive the energy intensity figures are outlined in further detail in the methods section. The derived county energy intensities in more detail can be found in the county datasets published on Zenodo (see Appendix B).

**Table 9:** County Disaggregated Energy Balance (TJ) for the base year of 2019, including aggregated energy consumption data for the agriculture, construction, mining, manufacturing, household and services sectors.

County Resolution Energy Balance 2019 TJ							
County	Agriculture	Construction	Mining	Manufacturing	Services	Household	Total
Baringo	4.925	11.667	0.893	33.747	3.494	755.303	810.029
Bomet	14.857	70.023	4.407	78.227	3.6683	1021.525	1192.706
Bungoma	16.236	35.917	2.108	59.491	8.517	1892.407	2014.676
Busia	5.811	51.081	2.095	21.18	3.757	1012.354	1096.279
Elgeyo Marakwet	13.949	6.819	3.448	18.467	2.678	514.831	560.194
Embu	7.342	77.507	1.616	24.612	7.502	689.416	807.994
Garissa	3.1501	13.737	3.881	9.854	3.026	953.077	986.727
Homa Bay	9.559	15.766	3.027	39.869	5.091	689.416	762.728
Isiolo	0.834	17.424	0.282	3.504	1.537	303.5903	327.17
Kajiado	3.868	85.733	19.544	59.744	8.642	1266.279	1443.809
Kakamega	15.705	22.109	10.245	110.629	9.126	2115.577	2283.392
Kericho	13.668	30.685	1.038	86.658	5.868	991.973	1129.89
Kiambu	17.062	545.738	29.204	543.119	25.314	2738.789	3899.228
Kilifi	7.955	13.877	23.681	296.449	9.948	1646.837	1998.748
Kirinyaga	11.244	4.323	10.12	41.885	5.134	691.468	764.175
Kisii	15.543	42.674	2.916	43.325	8.154	1264.689	1377.3005
Kisumu	7.731	108.402	11.617	216.632	13.275	1309.024	1666.682
Kitui	8.437	20.669	4.512	31.438	6.651	1287.063	1358.769
Kwale	6.9301	29.0905	64.937	48.735	4.602	98.193	252.486
Laikipia	4.699	54.082	0.808	19.788	4.673	587.4203	671.471
Lamu	1.905	0.958	0.893	9.586	1.824	163.031	178.198
Machakos	9.895	158.378	68.483	493.705	13.0303	1610.752	2354.243
Makueni	6.052	29.461	4.531	38.722	5.929	1118.805	1203.5
Mandera	3.5502	23.579	1.261	8.075	2.457	982.648	1021.569
Marsabit	2.563	72.926	0.512	4.689	2.805	520.8405	604.336
Meru	34.756	43.597	22.342	121.694	10.042	1750.971	1983.403
Migori	8.794	7.848	42.779	26.906	5.112	1435.088	1526.527
Mombasa	0.688	367.768	8.032	625.87	28.484	1368.789	2399.631
Murang’a	17.447	72.065	15.827	58.953	6.281	1196.953	1367.525
Nairobi	1.017	1749.961	5.365	2414.885	173.145	4980.966	9325.339
Nakuru	22.059	160.917	4.223	256.738	25.247	2449.324	2918.508
Nandi	16.021	9.813	4.209	64.5128	4.301	1003.326	1102.184
Narok	14.968	9.897	1.5104	59.878	6.226	1311.628	1404.109
Nyamira	10.538	24.949	9.779	39.125	3.902	685.991	774.285
Nyandarua	19.606	4.281	5.037	16.134	3.367	723.048	771.473
Nyeri	14.454	12.933	15.925	100.095	8.935	859.974	1012.316
Samburu	1.0005	6.197	0.335	9.649	1.795	351.536	370.513
Siaya	7.2545	41.072	8.071	24.605	4.5003	1125.069	1210.572
Taita Taveta	2.799	19.396	5.168	19.7801	3.491	385.909	436.544
Tana River	1.798	1.063	0.401	5.0301	1.521	357.897	367.711
Tharaka Nithi	3.635	31.336	1.274	21.844	2.623	445.387	506.1003
Trans Nzoia	12.018	24.586	1.937	43.0402	7.034	1121.849	1210.465
Turkana	6.569	30.273	9.253	16.712	5.328	1050.0704	1118.205
Uasin-Gishu	10.933	68.372	5.063	94.519	12.566	1317.647	1509.1002
Vihiga	6.434	37.764	11.841	10.661	3.197	668.3616	738.258
Wajir	3.292	28.999	3.192	9.158	2.157	885.008	931.808
West Pokot	5.113	4.042	20.128	7.007	3.305	703.736	743.333
Total	434.67	4299.759	477.751	6388.93	489.26	52403.84	64494.21

**Table 10:** Derived energy intensity figures (MJ/Kenyan Shillings) for each fuel type (motive, electricity, and thermal) per sector (agriculture, construction, mining, manufacturing, and services) per county.

County	Agriculture	Construction			Mining			Manufacturing		Services
	Motive	Electricity	Motive	Thermal	Electricity	Motive	Thermal	Electricity	Thermal	Electricity
Baringo	0.000202	0.002406	0.004123	0.000204	0.002287	0.003921	0.000194	0.000072	0.003886	0.000087
Bomet	0.000201	0.002416	0.004139	0.000204	0.002255	0.003865	0.000191	0.000072	0.003853	0.000087
Bungoma	0.000202	0.00243	0.004165	0.000206	0.002223	0.00381	0.000188	0.000072	0.003877	0.000087
Busia	0.000203	0.002441	0.004183	0.000207	0.00231	0.003959	0.000196	0.000072	0.003887	0.000087
Elgeyo Marakwet	0.000201	0.002413	0.004135	0.000204	0.002269	0.003888	0.000192	0.000071	0.003843	0.000086
Embu	0.000201	0.002423	0.004152	0.000205	0.002239	0.003837	0.000189	0.000072	0.003865	0.000106
Garissa	0.000203	0.002445	0.004191	0.000207	0.002283	0.003914	0.000193	0.000072	0.00389	0.000088
Homa Bay	0.000203	0.002438	0.004179	0.000206	0.002294	0.003932	0.000194	0.000072	0.00389	0.000087
Isiolo	0.0002	0.002414	0.004137	0.000204	0.002283	0.003913	0.000193	0.000071	0.003859	0.000087
Kajiado	0.000201	0.002409	0.004128	0.000204	0.002249	0.003855	0.00019	0.000071	0.003838	0.000087
Kakamega	0.000201	0.002417	0.004142	0.000205	0.002254	0.003863	0.000191	0.000072	0.003853	0.000086
Kericho	0.000202	0.002423	0.004152	0.000205	0.002359	0.004044	0.001997	0.000071	0.003865	0.000088
Kiambu	0.000199	0.002403	0.004118	0.000203	0.002243	0.003844	0.000189	0.000071	0.00383	0.000088
Kilifi	0.000203	0.002457	0.00421	0.000208	0.005124	0.008782	0.000434	0.000072	0.003893	0.000086
Kirinyaga	0.000213	0.002616	0.004483	0.000221	0.002401	0.004114	0.000203	0.000076	0.004093	0.000092
Kisii	0.000201	0.002425	0.004155	0.000205	0.002271	0.003892	0.000192	0.000072	0.003859	0.000086
Kisumu	0.000203	0.002447	0.004194	0.000207	0.0023	0.003942	0.000195	0.000072	0.003899	0.000089
Kitui	0.000181	0.002172	0.003722	0.000184	0.002037	0.003491	0.000172	0.000064	0.003464	0.000077
Kwale	0.000201	0.002417	0.004142	0.000205	0.00227	0.003892	0.000192	0.000071	0.003857	0.000087
Laikipia	0.0002	0.002409	0.004129	0.000204	0.002247	0.00385	0.00019	0.000071	0.003894	0.000086
Lamu	0.000201	0.00244	0.004182	0.002065	0.002275	0.003898	0.000193	0.000072	0.003856	0.000086
Machakos	0.000201	0.002424	0.004155	0.000205	0.002273	0.003895	0.000192	0.000072	0.003864	0.000088
Makueni	0.000201	0.002424	0.004154	0.000205	0.002278	0.003905	0.000192	0.000072	0.003861	0.000086
Mandera	0.000205	0.002468	0.00423	0.000209	0.002355	0.004035	0.000199	0.000073	0.003954	0.000089
Marsabit	0.000202	0.002424	0.004156	0.000205	0.002236	0.003832	0.000189	0.000071	0.003842	0.000138
Meru	0.000206	0.002483	0.004556	0.00021	0.002321	0.003978	0.000196	0.000073	0.003954	0.000089
Migori	0.000201	0.002416	0.004139	0.000204	0.002272	0.003894	0.000192	0.000072	0.003867	0.000087
Mombasa	0.000199	0.0024009	0.004114	0.000203	0.002256	0.003867	0.000191	0.000071	0.003826	0.000088
Murang’a	0.000201	0.002416	0.004141	0.000205	0.002271	0.003891	0.000192	0.000071	0.003849	0.000088
Nairobi	0.000195	0.002395	0.004106	0.000203	0.002368	0.004058	0.0002	0.000071	0.003821	0.000086
Nakuru	0.0002	0.002409	0.004128	0.000204	0.002248	0.003853	0.00019	0.000071	0.003838	0.000103
Nandi	0.000203	0.002467	0.004227	0.000209	0.002277	0.003903	0.000193	0.000072	0.003903	0.000088
Narok	0.000201	0.002424	0.004154	0.000205	0.002219	0.003803	0.000188	0.000072	0.003853	0.000086
Nyamira	0.000202	0.002437	0.004177	0.000206	0.002277	0.003903	0.000193	0.000072	0.00388	0.000087
Nyandarua	0.000201	0.00244	0.004182	0.000207	0.002287	0.003919	0.000194	0.000071	0.003467	0.000086
Nyeri	0.000201	0.002424	0.004155	0.000205	0.002268	0.003888	0.000192	0.000071	0.003843	0.000086
Samburu	0.000202	0.002424	0.004153	0.000205	0.002292	0.003928	0.000194	0.000072	0.003871	0.000086
Siaya	0.000202	0.002432	0.004168	0.000206	0.002285	0.003917	0.000193	0.000072	0.003879	0.000087
Taita Taveta	0.0002	0.002413	0.004136	0.000204	0.002268	0.003887	0.000191	0.000071	0.003842	0.000086
Tana River	0.000204	0.002449	0.004198	0.000207	0.002307	0.003954	0.000195	0.000073	0.003908	0.000087
Tharaka Nithi	0.000202	0.002426	0.004158	0.000205	0.002309	0.003958	0.000195	0.000072	0.003866	0.000086
Trans Nzoia	0.000201	0.002415	0.004139	0.000204	0.002324	0.003983	0.000197	0.000072	0.003853	0.000086
Turkana	0.000203	0.002553	0.004375	0.000216	0.002289	0.003924	0.000194	0.000072	0.003897	0.000086
Uasin-Gishu	0.000201	0.002412	0.004134	0.000204	0.002291	0.003926	0.000194	0.000071	0.003847	0.000086
Vihiga	0.000203	0.002448	0.004195	0.000207	0.002296	0.003934	0.000194	0.000072	0.003892	0.000088
Wajir	0.000202	0.002435	0.004174	0.000206	0.002294	0.003932	0.000194	0.000072	0.003881	0.000087
West Pokot	0.000201	0.002425	0.004155	0.000205	0.002274	0.003896	0.000192	0.000072	0.003859	0.000096

**Table 11:** Derived energy intensity figures (MJ/Dwellings/Year) for urban and rural households, per end use (cooking lighting and appliances) per fuel type (fossil fuels and appliances).

County	Urban				Rural			
	Cooking	Lighting Electricity	Lighting Fossil Fuels	Appliances Electricity	Cooking	Lighting Electricity	Lighting Fossil Fuels	Appliances Electricity
Baringo	14566.91	489.129	56.095	440.216	14566.91	489.129	56.095	440.216
Bomet	14566.86	489.128	56.095	440.215	14566.86	489.128	56.095	440.215
Bungoma	14256.97	478.72	54.902	430.85	14256.97	478.72	54.902	430.85
Busia	13947.11	468.32	53.71	421.49	13947.11	468.32	53.71	421.49
Elgeyo Marakwet	13947.04	468.315	53.708	421.484	13947.04	468.315	53.708	421.484
Embu	10227.82	343.43	39.39	309.088	10227.82	343.43	39.39	309.088
Garissa	18286.12	614.01	70.42	552.61	18286.12	614.01	70.42	552.61
Homa Bay	7165.42	240.6	27.593	216.54	7165.42	240.6	27.593	216.54
Isiolo	14256.97	478.72	54.902	430.85	14256.97	478.72	54.902	430.85
Kajiado	10847.7	364.245	41.773	327.821	10847.7	364.245	41.773	327.821
Kakamega	13327.17	447.501	51.321	402.751	13327.17	447.501	51.321	402.751
Kericho	13637.1	457.908	52.515	412.117	13637.1	457.908	52.515	412.117
Kiambu	9298.025	312.21	35.805	280.989	9298.025	312.21	35.805	280.989
Kilifi	14876.84	499.54	57.29	449.58	14876.84	499.54	57.29	449.58
Kirinyaga	9298.023	312.21	35.805	280.989	9298.023	312.21	35.805	280.989
Kisii	12707.3	426.687	48.934	384.019	12707.3	426.687	48.934	384.019
Kisumu	11777.54	395.47	45.354	355.921	11777.54	395.47	45.354	355.921
Kitui	13327.17	447.501	51.321	402.75	13327.17	447.501	51.321	402.75
Kwale	1549.67	52.035	5.968	46.832	1549.67	52.035	5.968	46.832
Laikipia	10537.76	353.838	40.579	318.454	10537.76	353.838	40.579	318.454
Lamu	11467.56	385.059	44.16	346.553	11467.56	385.059	44.16	346.553
Machakos	10847.69	364.25	41.773	327.821	10847.69	364.25	41.773	327.821
Makueni	12397.37	416.741	47.741	374.65	12397.37	416.741	47.741	374.65
Mandera	21385.46	718.08	82.35	646.28	21385.46	718.08	82.35	646.28
Marsabit	17976.18	603.606	69.224	543.246	17976.18	603.606	69.224	543.246
Meru	11157.63	374.65	42.967	337.187	11157.63	374.65	42.967	337.187
Migori	14256.97	478.722	54.902	430.85	14256.97	478.722	54.902	430.85
Mombasa	9706.96	322.62	36.99	290.36	9706.96	322.62	36.99	290.36
Murang’a	10227.83	343.43	39.386	309.088	10227.83	343.43	39.386	309.088
Nairobi	8988.09	301.803	34.612	271.623	8988.09	301.803	34.612	271.623
Nakuru	10847.7	364.245	41.773	327.821	10847.7	364.245	41.773	327.821
Nandi	13637.1	457.908	52.515	412.117	13637.1	457.908	52.515	412.117
Narok	14876.84	499.536	57.289	449.583	14876.84	499.536	57.289	449.583
Nyamira	12397.37	416.28	47.74	374.65	12397.37	416.28	47.74	374.65
Nyandarua	10847.7	364.25	41.77	327.82	10847.7	364.25	41.77	327.82
Nyeri	9298.02	312.21	35.81	280.99	9298.02	312.21	35.81	280.99
Samburu	14566.91	489.13	56.095	440.216	14566.91	489.13	56.095	440.216
Siaya	12087.43	405.87	46.547	365.29	12087.43	405.87	46.547	365.29
Taita Taveta	10847.73	364.25	41.773	327.822	10847.73	364.25	41.773	327.822
Tana River	14256.97	478.72	54.902	430.85	14256.97	478.72	54.902	430.85
Tharaka Nithi	4488.57	150.72	17.285	135.646	4488.57	150.72	17.285	135.646
Trans Nzoia	13637.11	457.908	52.515	412.118	13637.11	457.908	52.515	412.118
Turkana	17356.31	582.792	66.837	524.513	17356.31	582.792	66.837	524.513
Uasin-Gishu	11777.5	395.47	45.354	355.919	11777.5	395.47	45.354	355.919
Vihiga	12707.37	426.689	48.934	384.02	12707.37	426.689	48.934	384.02
Wajir	18905.98	634.827	72.804	571.344	18905.98	634.827	72.804	571.344
West Pokot	16426.51	551.571	63.256	496.414	16426.51	551.571	63.256	496.414

## 4 Experimental Design, Materials and Methods

This section outlines the sources and processing methods used for the socio-economic and technical parameters within this dataset. The data was collected via publically available sources, including the reports of national and international organisations, journal articles, and existing databases, complying with the U4RIA analytics and good governance goals of Ubuntu, Retrievability, Reusability, Repeatability, Reconstructability, Interoperability, and Auditability. The U4RIA goals aim to provide guidance on best practices within energy modelling [14]. The datasets include raw data on gross county product (GCP), population alongside national energy consumption, energy efficiency and electrification rates where available. The data in the County Demand Starter Data Kit were collected from the KNBS data portal [5], economic [7,9,15,16] and census [3,12,[17], [18], [19]] reports, the KIPPRA economic report [8], the International Energy Agency (IEA) world energy balances [10], the commission on revenue allocation county fact sheets [13] and previous modelling literature [11]. Due to missing data on county and sectoral disaggregated energy balances, assumptions were made by the authors to address this. Once the raw data was collected, a systemic procedure of organisation, standardisation and analysis was undertaken to meet the modelling input parameters. All data was screened and crosschecked with alternative datasets if any errors or inconsistencies were identified. The data were collected for use with the Model for the Analysis of Energy Demand (MAED) tool, which can project whole energy system demand based on historical data. Despite this, the data outlined through this document remains independent from the models and tools identified. The time-period (2019-2070) is selected based on data availability, for the base year of 2019, and relevancy to long-term energy planning time scales in national Kenyan energy planning.

### 4.1 Population

To acquire the input data for the population parameters, historic data was collected for the base year of 2019, found in Table 3. Existing population growth % projections were collected from the KNBS data portal [8] at a county resolution from 2019 to 2045, with additional projections from 2045 to 2070 assumed to follow the decreasing population growth rate trends identified in the previous half of the modelling period. The complete population growth rate inputs from 2019 to 2070 across the counties can be accessed via the Appendix Table B1. Due to a lack of available data on projections for urban/rural split, historic average urbanisation rates from the world bank database [4] were identified as a 0.5% increase annually and was assumed to remain at this rate across the modelling period. Due to an absence of projections for average household size the values are assumed to remain constant across the modelling period. Additionally, average household sizes were assumed to be the same for urban and rural households due to the absence of disaggregated data.

### 4.2 Economic

To acquire the input data for the population parameters, historic data was collected for the base year of 2019, found in Table 4. Due to a lack of available data, the GCP growth rates (Fig. 2) were based on the 2014–2017 GCP growth rate average %, taken from the 2020 KIPPRA economic report, and assumed to remain constant across the modelling period of 2019 to 2070 [8]. Similarly, data on the sectoral percentage contribution to total GCP was extracted from the KNBS 2021 Gross County Product report [9] and assumed to remain the same across the 2019 to 2070 modelling period.

### 4.3 Electrification

Data on county electrification rates alongside average annual increase in electrification from 2009 to 2019 were obtained from the commission of revenue allocations Kenya county fact sheets third edition [13]. Electrification rates are assumed to increase annually in line with the average rates from the last decade. Due to a lack of urban and rural disaggregated data at a county resolution, the data is assumed to be the same.

### 4.4 Energy balances

**Fig. 7:**
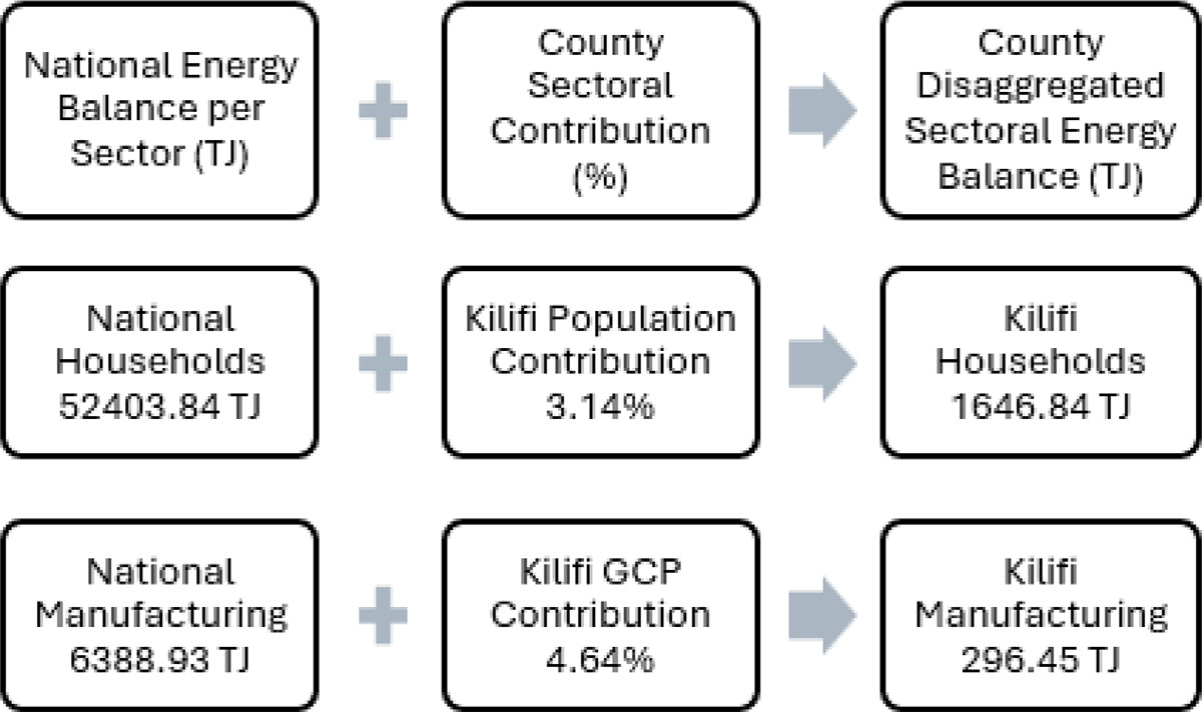
An illustrative representation of the downscaling methodology employed to acquire county resolution energy balance data from raw national energy balance data using the example of household and manufacturing sectors for Kilifi. Note that the household sector is disaggregated based on the % of the national population for Kenya which resides in Kilifi whereas the manufacturing sector is disaggregated based on % contribution of sectors GCP to total national GDP.

**Fig. 8:**
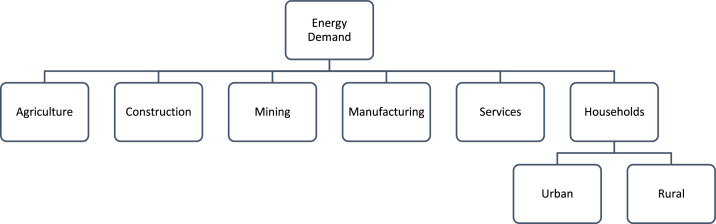
An illustrative overview of the demand structure employed in the Model of the Analysis of Energy Demand (MAED).

The county resolution energy balances were processed using a three-step methodology consisting of: [1] collecting a raw national energy balance, [2] processing the national data collected to fit into the format and demand structure required for MAED, and [3] disaggregating the national energy balance to a county scale. For the second step, the energy balance fuel consumption data was aggregated into fossil fuels (bitumen, LPG, motor gas, kerosene, gas and diesel oil, fuel oil), traditional fuels (biofuels and charcoal) and electricity to fit the MAED sectoral and fuel categorisations. Additionally, due to a lack of data, industry (non-specified) energy consumption was split into construction and mining proportionate to the sector contribution to total national GDP as obtained from the KIPPRA Kenya Economic Report 2020 [8]. It is therefore assumed that construction consumed 90 % and mining 10 % of the non-specified industrial consumption. For the second step, due to a lack of accessible energy balance data at a county resolution, the 2019 energy consumption data have been disaggregated from the national data. Energy consumption for the economic sectors (agriculture, mining, manufacturing, construction and services) has been disaggregated proportionally to the counties’ individual sectoral contribution to the total national sectoral GDP. Household energy consumption has been disaggregated proportionally to the percentage of the total population residing in the counties compared to national figures. Fig. 7 outlines the methodology utilised for the energy balance disaggregation, using the case study of Kilifi’s household and manufacturing sectors as an example. This disaggregation was constructed to align with the structure of the Model for the Analysis of Energy Demand (MAED) tool (Fig. 8), however the data outline in this paper remains independent from the tool.

### 4.5 Energy intensities

**Fig. 9:**
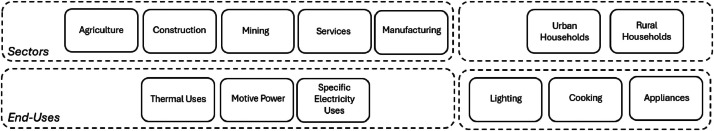
An illustrative overview of the MAED energy intensity structure for individual energy end uses per sector. Note that the agriculture, construction, mining, manufacturing and services sectors and disaggregated into the end uses of thermal, motive and electricity. Whereas rural and urban households are disaggregated by the end uses of lighting, cooking and appliances.

Energy intensity figures for individual energy end-uses per sector were derived in line with the MAED demand structure (Fig. 9). For all the sectors, due to a lack of available county resolution data on energy consumption by end use per sector, assumptions on the proportional split were made in line with the national OSeMOSYS demand workbook [11], for agriculture, manufacturing and services, and Kiley [2] for construction and mining. Such assumptions were used alongside the driving parameters identified in the population and economics sections to acquire the energy intensity figures to be used as technical inputs for the demand modelling. Energy intensity figures for agriculture, construction, mining, manufacturing, and services were derived as MJs per sectoral Kenyan Shilling. For household energy intensities, due to a lack of urban and rural disaggregated data at a county resolution, the energy intensity figures are assumed to be the same. Household energy intensity figures were derived as MJs per household per year.

### 4.6 Potential future updates

Through consultation with key stakeholders within Kenya, both county and national energy planning units have expressed interest in the application and utilisation of the datasets. Therefore, whilst the datasets have not been applied in any research so far, the authors hope, and have plans for, the co-production of county energy planning studies such as investigations into forecasted whole energy system demand and the development of capacity expansion plans for county case studies in the present future. Additionally, the authors hope to use the starter data kits and base MAED models produced to undertake capacity building and teaching workshops for both county and national energy planning units in the future. The authors are also optimistic that this work can play a part in starting to build greater county-national dialogue and data exchange in energy planning within Kenya.

The authors plan to build upon and expand the data sets produced within this iteration of the demand starter kits, updating the datasets following any additional publications and data collection completed on the ground in the counties. Particularly, the incorporation of bottom-up energy balance data, as opposed to relying on national disaggregation methodologies should serve to strengthen the datasets in the future. Additionally, the integration of specific energy demand profile needs for gender and social inclusion (GESI), resilience and climate adaptation considerations are also being considered for future demand kit development.

## Limitations

As shown throughout the dataset, available open-access socio-economic and technical data at a county resolution in Kenya is challenging to find and limited in availability. Consequently, this dataset relies on various assumptions, as identified in the methods section, to overcome data unavailability issues. Particularly, the datasets assumes that county energy consumption is proportional to economic and population activity to acquire the county resolution energy balances. Additional assumptions, beast on best available data, were employed to acquire end consumption by end uses within the sectors as outlined in the methodology section. Whilst these assumptions may produce disaggregate energy balances which do not fully reflect reality, the absence of existing data means that such assumptions were necessary. Additionally, some of the data utilised from international and national databases are often inconsistent and based themselves heavily on assumptions or unreliable sources. Particularly, the census data sources used within this study have been criticised for inconsistent and unscientific methodologies, leading to unrepresentative data for the counties of Garissa, Mandera, and Wajir. Subsequently such census data has been revoked and a new collection ordered. However, this paper publishes these data regardless due to a current lack of available alternative data, considering the last census data was published in 2009, and offers this data as a starting point for further data to be collected and built upon, alongside capacity building and teaching efforts to be undertaken for the 47 counties in Kenya listed in the Appendix Table B1. The authors acknowledge that whilst the population data for the three counties are unrepresentative, they offer the best available data and would be more representative than the previous 2009 data. Additionally, the authors plan to update the data kits for the three outlined counties as soon as the revised census is undertaken and data published.

## Ethics Statement

The authors have read and followed the ethical requirements for publication in Data in Brief and confirm that the current work does not involve human subjects, animal experiments, or any data collected from social media platforms.

## CRediT Author Statement

**Neve Fields:** Conceptualisation, Methodology, Data Curation, Writing – Original draft preparation, validation, visualisation, Writing – reviewing and editing. **Ariane Millot**: Conceptualisation, Methodology, Data Curation. **Pietro Lubello**: Conceptualisation, Data Curation. **Martin Mutembei**: Investigation. **Anne Nganga:** Investigation. **Leonard Hofbauer**: Conceptualisation. **Mark Howells**: Supervision. **Ed Brown**: Supervision.

## Figures and Tables

**Fig. A1:**
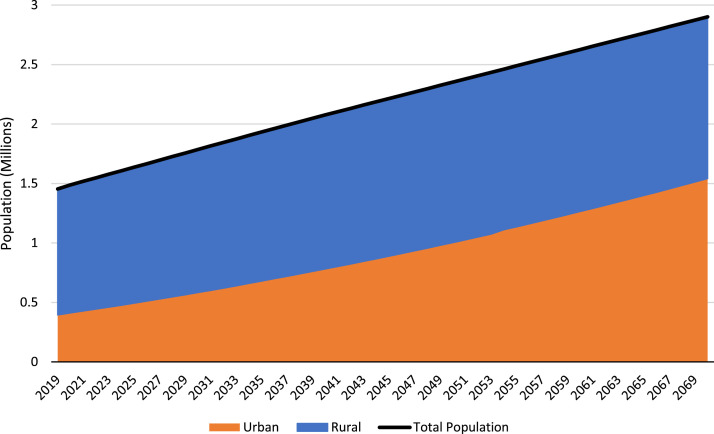
Total population (million people) projection disaggregated by urban and rural in Kilifi County from 2019 to 2070.

**Fig. A2:**
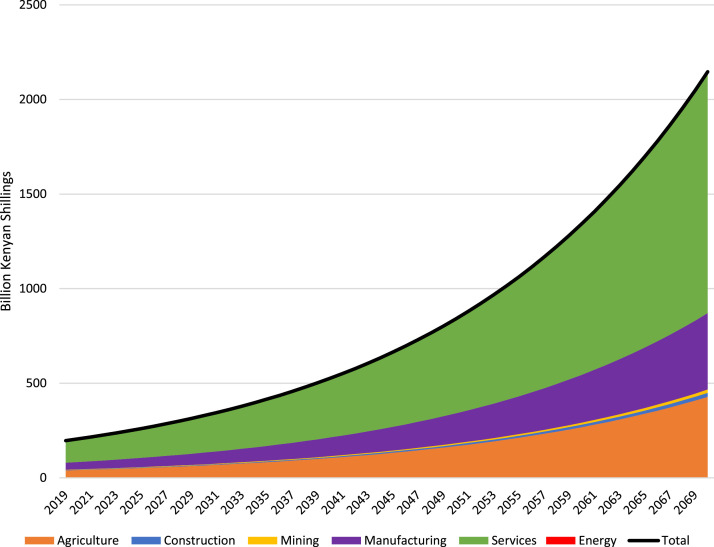
Total GCP (Billion Kenyan Shillings) projection in Kilifi County disaggregated by sector from 2019 to 2070.

**Fig. A3:**
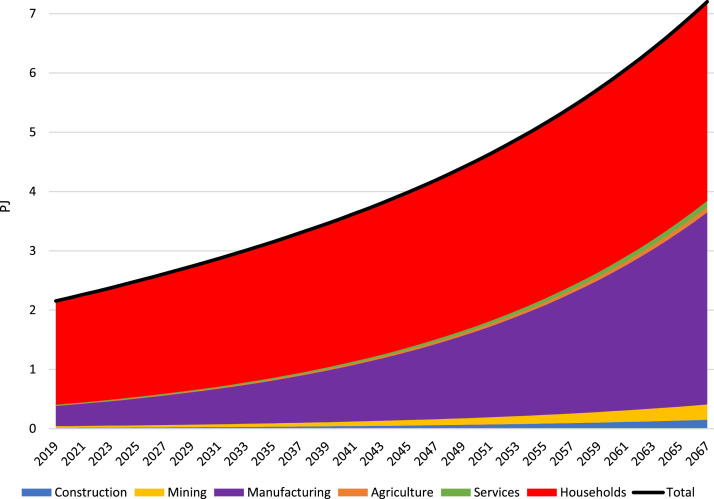
A possible baseline demand projection for Kilifi County from 2019 to 2070 using template energy data.

**Table B1:** lists the county-specific datasets that were produced utilising the data outlined within this article. For each county, there is an associated dataset which includes the data in a CVS, a pre-print article which describes the data collection process and illustrates an example demand projection created utilising the MAED model, and the accompanying MAED model. These can act as the basis for county-level analysis and further future model development.

County	Zenodo Repository Link
All	10.5281/zenodo.14725146
Baringo	10.5281/zenodo.14724131
Bomet	10.5281/zenodo.14724284
Bungoma	10.5281/zenodo.14724344
Busia	10.5281/zenodo.14724355
Elgeyo Marakwet	10.5281/zenodo.14724093
Embu	10.5281/zenodo.14719694
Garissa	10.5281/zenodo.14719587
Homa Bay	10.5281/zenodo.14724419
Isiolo	10.5281/zenodo.14719658
Kajiado	10.5281/zenodo.14724248
Kakamega	10.5281/zenodo.14724301
Kericho	10.5281/zenodo.14724268
Kiambu	10.5281/zenodo.14723995
Kilifi	10.5281/zenodo.14719477
Kirinyaga	10.5281/zenodo.14719819
Kisii	10.5281/zenodo.14724455
Kisumu	10.5281/zenodo.14724394
Kitui	10.5281/zenodo.14719710
Kwale	10.5281/zenodo.14719340
Laikipia	10.5281/zenodo.14724157
Lamu	10.5281/zenodo.14719518
Machakos	10.5281/zenodo.14719741
Makueni	10.5281/zenodo.14719756
Mandera	10.5281/zenodo.14719621
Marsabit	10.5281/zenodo.14748987
Meru	10.5281/zenodo.14719666
Migori	10.5281/zenodo.14724433
Mombasa	10.5281/zenodo.14719306
Murang’a	10.5281/zenodo.14723976
Nairobi	10.5281/zenodo.14724484
Nakuru	10.5281/zenodo.14724176
Nandi	10.5281/zenodo.14724107
Narok	10.5281/zenodo.14724194
Nyamira	10.5281/zenodo.14724467
Nyandarua	10.5281/zenodo.14719765
Nyeri	10.5281/zenodo.14719795
Samburu	10.5281/zenodo.14724048
Siaya	10.5281/zenodo.14724371
Taita Taveta	10.5281/zenodo.14719540
Tana River	10.5281/zenodo.14719498
Tharaka Nithi	10.5281/zenodo.14719679
Trans Nzoia	10.5281/zenodo.14724066
Turkana	10.5281/zenodo.14724013
Uasin-Gishu	10.5281/zenodo.14724073
Vihiga	10.5281/zenodo.14724315
Wajir	10.5281/zenodo.14719608
West Pokot	10.5281/zenodo.14724035

## Data Availability

ZenodoDemand Starter Data Kit: Selected socio-economic and technical energy system demand modelling data for all 47 counties in Kenya. (Original data)

## Appendix A – Zero**-order Basic MAED Model**

The data described above were used to create a simple demand projection model for Kilifi County Kenya. As it is open-source and free, a MAED model is calibrated and ran with one baseline example projection. This model and the results are intended to provide an example of what could be produced through applying the data outlined in this article and therefore acts as a starting point for further model development. The data described in this article were used as input data to define assumptions in the model Fig. A1, Fig. A2, Fig. A3.

### A1 Model assumptions

To produce a generic baseline demand projection model for Kilifi, various assumptions were introduced. For the demographic parameters, it is assumed that average urban and rural household sizes will remains the same for the duration of the modelling period. An average annual urbanisation rate of 0.5 % is assumed throughout the modelling period. In the economic parameters, the sectoral shares of GCP and the average annual GCP growth rates are assumed to remain constant across the modelling period. In the agricultural sector, 100 % of the total fossil fuel is assigned to motive power. In the construction and mining sectors, 90 % of the fossil fuels is allocated to motive power and the remaining 10 % to thermal uses, as well as 100 % of the electricity as specific electricity use. For the manufacturing sector, 100 % of the electricity is assigned as specific electricity usage, and 90 % of the fossil fuels assumed to be low level thermal usage and 10 % high level thermal usage. In the services sector, 100 % of the electricity consumed is assumed to be for specific electricity use. Finally, in the household sector, for fossil fuels 95 % is assigned to cooking demand and 5 % to lighting, for traditional fuels 100 % is allocated to cooking and for electricity 5 % to cooking, 50 % to lighting and 45 % to electrical appliances.

### A2 Model Results for Kilifi County

The graphs below show selected results from the model application, including an overview of the population and economic assumptions alongside the produced demand projection from 2019 to 2070.

The examples of possible results were achieved through applying the data outlined in this article to MAED. Those interested in further development of demand modelling through the application of this work and recommended to access the extended comprehensive individual county datasets (see Appendix B) and guidance on model development using MAED [*23*].

## Appendix B – **County Datasets**

Table B1
